# Supplementary material for: Electrically programmable magnetic coupling in an Ising network exploiting solid-state ionic gating
Source: Nat Commun. 2023 Oct 11;14:6367. doi: 10.1038/s41467-023-41830-5 (PMC10567909; doi:10.1038/s41467-023-41830-5)
Supplement: Supplementary file 1 — Supplementary Information [file 41467_2023_41830_MOESM1_ESM.docx]

**Supplementary Information**

**for**

**Electrically programmable magnetic coupling in an Ising network**

**exploiting solid-state ionic gating**

Chao Yun^1,2†^, Zhongyu Liang^1†^, Aleš Hrabec^3,4,5^, Zhentao Liu^3,4^, Mantao Huang^6^,

Leran Wang^1^, Yifei Xiao^7^, Yikun Fang^7^, Wei Li^7^, Wenyun Yang^1^, Yanglong Hou^2^, Jinbo Yang^1^,

Laura J. Heyderman^3,4*^, Pietro Gambardella^5*^, Zhaochu Luo^1*^

^1^State Key Laboratory of Artificial Microstructure and Mesoscopic Physics, School of Physics, Peking University, 100871 Beijing, China.

^2^School of Materials Science and Engineering, Peking University, 100871 Beijing, China.

^3^Laboratory for Mesoscopic Systems, Department of Materials, ETH Zurich, 8093 Zurich, Switzerland.

^4^Laboratory for Multiscale Materials Experiments, Paul Scherrer Institute, 5232 Villigen PSI, Switzerland.

^5^Laboratory for Magnetism and Interface Physics, Department of Materials, ETH Zurich, 8093 Zurich, Switzerland.

^6^Department of Materials Science and Engineering, Massachusetts Institute of Technology, Cambridge, MA, USA

^7^Division of functional Materials, Central Iron and Steel Research Institute Group, 100081 Beijing, China.

†These authors contributed equally: Chao Yun, Zhongyu Liang

*Correspondence to: [zhaochu.luo@pku.edu.cn](mailto:zhaochu.luo@pku.edu.cn) (Z.Luo); [pietro.gambardella@mat.ethz.ch](mailto:pietro.gambardella@mat.ethz.ch) (P.G.); [laura.heyderman@psi.ch](mailto:laura.heyderman@psi.ch) (L.J.H.).

S1. Device fabrication and magnetic characterization

S2. Demagnetization protocol

S3. Details of macrospin and semi-micromagnetic model

S4. Micromagnetic simulations for different *K*_g_

S5. Interplay between DMI and magnetic anisotropy

S6. Reliability of <*S_i_S_i_*_+1_> obtained from different chips and positions

S7. Effect of dipolar interaction

S8. Programmable coupling configuration in a four-spin chain

S9. Programmable Ising networks for the 8- and 10-vertex Max-Cut problems

S10. Hybrid MTJ/Ising network structure for general Ising computing

S11. Reconfigurable nanomagnetic logic gates

**S1. Device fabrication and magnetic characterization**

**
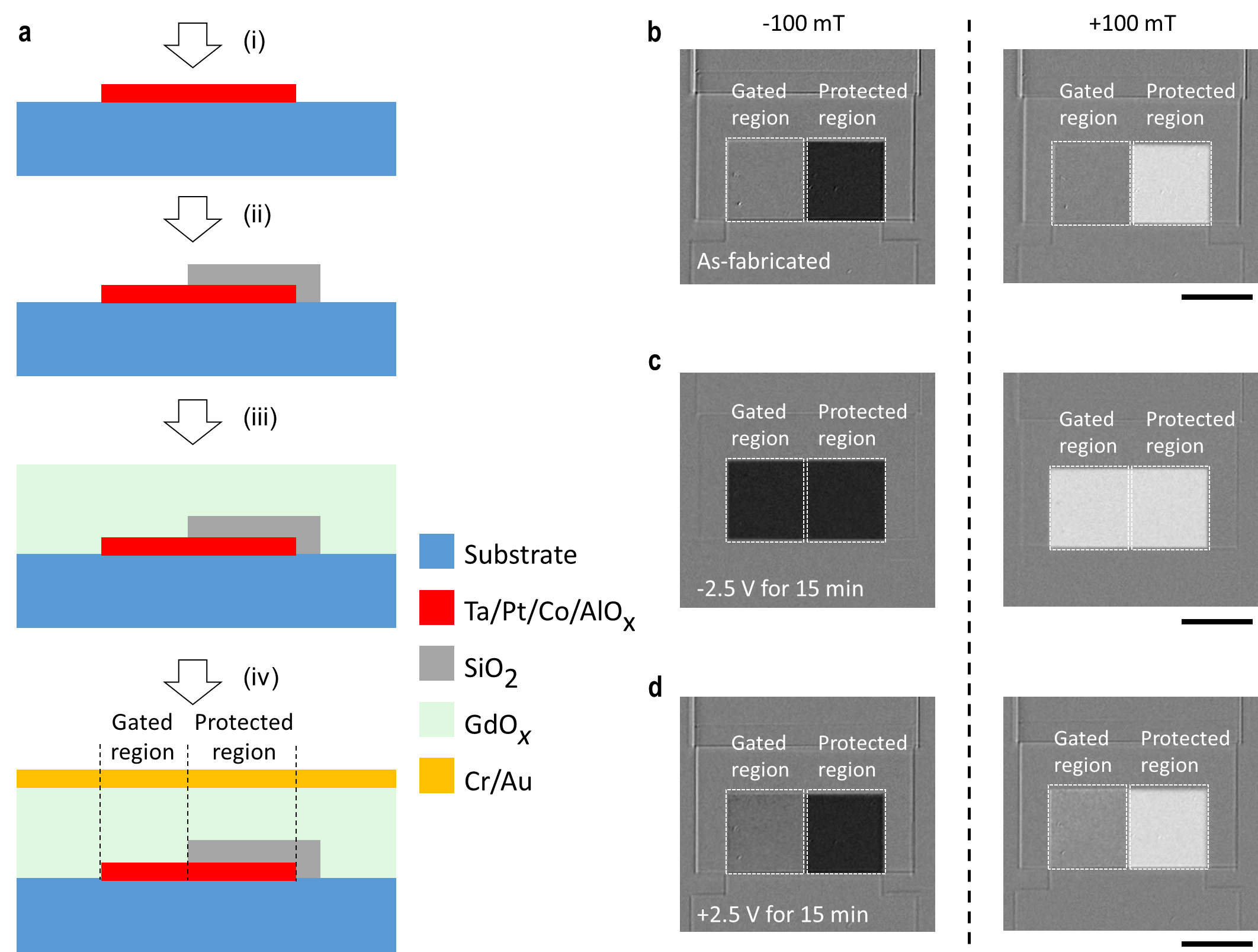
**

**Figure S1 | Device fabrication and magnetic characterization. a**, Schematic of main nanofabrication steps to create the electrically programmable coupled nanomagnets. (i) Ion milling of magnetic Ta (5 nm)/Pt (5 nm)/Co (1.5 nm)/AlO_x_ (2 nm) multilayer, (ii) deposition and lift off to create a patterned protective layer of Cr (2 nm)/SiO_2_ (8 nm), (iii) deposition of electrolyte layer of GdO_x_ (30 nm) and (iv) deposition and lift-off to create a gate electrode of Cr (2 nm)/Au (3 nm). **b** to **d**, Polar MOKE images showing the evolution of OOP magnetic anisotropy in the gated and protected regions on application of a gate voltage: the as-fabricated state (**b**), after applying -2.5 V for 15 min (**c**) and after applying +2.5 V for 15 min (**d**). Each MOKE image is captured after saturating the sample with OOP magnetic fields of -100 mT (left) and 100 mT (right), and is subtracted from the image captured at saturation magnetic fields of 100 mT (left) and -100 mT (right). The white, grey and black contrast in the magnetic regions corresponds to ↑, IP and ↓ magnetization, respectively. All scale bars are 20 μm.

**S2. Demagnetization protocol**

In order to obtain the low-energy magnetic configuration in an array of coupled nanomagnets, an oscillating magnetic field is applied perpendicular to the devices and the field amplitude is reduced over time (Fig. S2a). For this demagnetization protocol, the oscillating frequency of the magnetic field is 2 Hz (oscillation period *t*_0_ = 0.5 s) and its amplitude is linearly reduced from *H*_max_ = 200 mT to zero with a constant step size of Δ*H*_Demag_ = 0.167 mT. As shown in the MFM image of as-fabricated magnetic configuration of the Ising square-lattice element prior to applying magnetic fields (Fig. S2b), AFM-like domains are spontaneously formed, indicating the presence of AP coupling in the as-fabricated state. Following application of the demagnetization protocol, the size of the AFM-like domains increases, indicating that the array of coupled nanomagnets has been driven to a lower-energy state (Fig. S2c). Interestingly, on repeating the same demagnetization process, the AFM domain pattern changes, implying that the formation of AFM-like domains is stochastic.


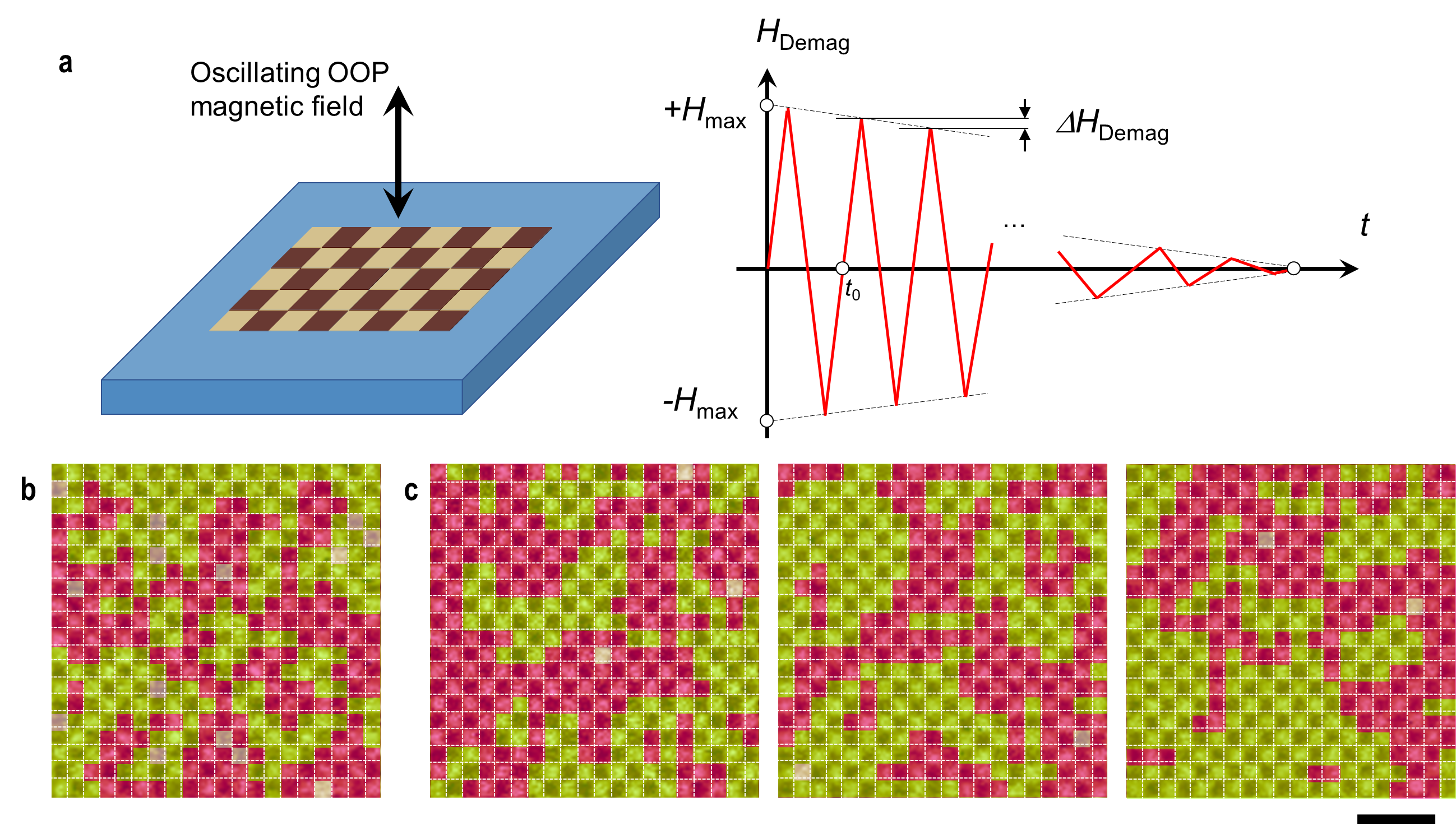


**Figure S2 | Demagnetization protocol. a**, Schematic showing the demagnetization procedure applied to the coupled nanomagnets. **b**, MFM image of the as-fabricated magnetic configuration of the Ising artificial spin ice prior to applying the demagnetization protocol. **c**, Three MFM images of the same area shown in **b** taken after repeating the same demagnetization protocol. AFM-like domains are shaded in green and red. The bright and dark contrast in the MFM images indicates nanomagnet regions with ↑ and ↓ magnetization, respectively. The scale bar is 1 µm.

To understand how the demagnetization protocol drives coupled nanomagnets to the low-energy state, we construct a square-lattice Ising macrospin model to simulate the demagnetization process. The macrospin approximation is used to model the thermally-active switching process with the switching probability *P*_SW_ given by^1^:

$P_{\text{sw}}=t_{\text{s}}f_{0}e^{-\frac{E_{\text{b}}}{kT}}$, (S1)

where *t*_s_, *f*_0_, *E*_b_, *k* and *T* represent the time to switch, the attempt frequency (~10^9^ Hz), the energy barrier to switching, the Boltzmann constant and temperature, respectively. The switching energy barrier can be determined using the Stoner-Wohlfarth model where:

, (S2)

when the effective magnetic field *H*_eff_ is parallel to the direction of the original magnetization and

, (S3)

when the effective magnetic field *H*_eff_ is antiparallel to the direction of the original magnetization. Here, the effective magnetic field *H*_eff_ includes the external magnetic field *H*_ext_ and the combined effect of the coupling with four nearest-neighbour sites:

$H_{\text{eff}}=H_{\text{ext}}+\sum_{\text{N.N.}} J/m$. (S4)

The magnetic moment *m* on the square-lattice site, the nearest-neighbour AP coupling strength *J* (-2.5 eV) and the anisotropy-induced switching energy barrier *E*_sw_ (15.7 eV) are all taken from the experimental results. Here, *J* and *E*_sw_ on each site is given by a Gaussian distribution to take into account the disorder in real devices. The magnetic configurations are obtained for different demagnetization step sizes Δ*H*_Demag_. The nearest-neighbour correlation <*S_i_S_i_*_+1_> is determined to evaluate how close the magnetic configuration is to the ground state. The AP ground state on the square lattice is well-defined, forming a “checkerboard” pattern with <*S_i_S_i_*_+1_> = -1. As shown in Fig. S3a, the magnetic configuration approaches the low-energy state on decreasing the demagnetization step size.

The demagnetization process can behave as a “thermal bath” that allows the array of coupled nanomagnets to relax into a low-energy configuration at an effective elevated temperature *T*_eff_ ^2,3^. We employ the Metropolis–Hastings algorithm to estimate *T*_eff_ for our demagnetization protocol using the same coupling strength and distribution as those used in the macrospin model. The change of <*S_i_S_i_*_+1_> with respect to the effective temperature parameter of *βJ* (*β* = 1/*kT*) is shown in Fig. S3b. A transition in <*S_i_S_i_*_+1_> occurs around *βJ* ≈ 0.5, which agrees with the theoretical prediction of phase transition in the square-lattice Ising model:

$\left(\beta J\right)_{\text{C}}=\frac{\ln(1+\sqrt{2})}{2}\approx0.44$. (S5)

By comparing the values of <*S_i_S_i_*_+1_> obtained using the macrospin model and using the Metropolis–Hastings algorithm, we can estimate the effectiveness of demagnetization protocol to drive the coupled nanomagnets to their ground state (Fig. S3c). The decrease of the demagnetization step size effectively decreases the temperature of “thermal bath” that saturates at a certain temperature (*ꞵJ* ≈ 0.44) related to disorder in the coupled system. It also verifies the effectiveness of our demagnetization protocol, with the experimental demagnetization step size of 0.167 mT being sufficient to realize the lowest-energy magnetic configuration.


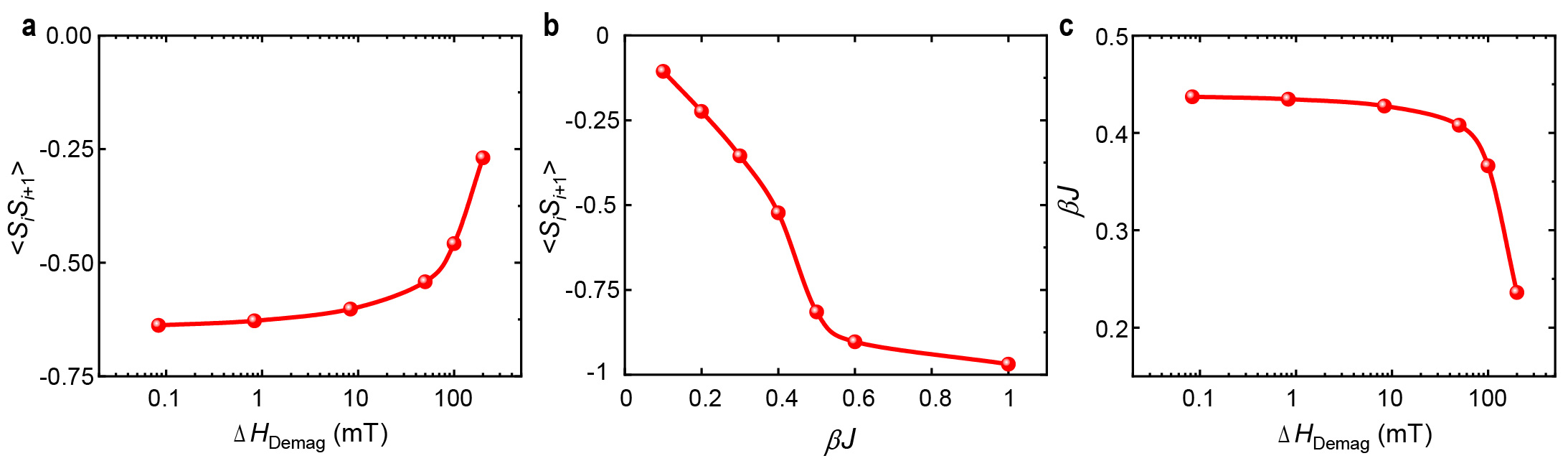


**Figure S3 | Simulation results of square-lattice Ising model. a**, <*S_i_S_i_*_+1_> as a function of the demagnetization step size Δ*H*_Demag_. **b**, <*S_i_S_i_*_+1_> as a function of the effective temperature *βJ* obtained with the Metropolis–Hastings algorithm for the square-lattice Ising model. **c**, Effective temperature parameter *βJ* as a function of the demagnetization step size Δ*H*_Demag_.

The protected regions are designed to have a high perpendicular magnetic anisotropy, which ensures that the magnetization is not perturbed by the stray field from the MFM magnetic tips during the measurements. This also means that the energy barrier for magnetization switching in the protected regions is higher than the thermal energy at room temperature. The coupled nanomagnet system is thus athermal and no thermally-active magnetization switching is observed during the experiments. Furthermore, the nearest-neighbour coupling strength is weaker than the energy barrier for switching the magnetization. Therefore, the voltage-controlled change of the coupling strength cannot induce the spontaneous switching of the magnetization without applying the demagnetization protocol.

As shown in Fig. S4a, the array of coupled nanomagnets exhibits an AFM-like pattern following demagnetization. The device was then exposed to a negative voltage of -2.5 V for 120 min, converting the coupling from AP to P. The same magnetic configuration was observed in the subsequent MFM measurement indicating that the energy barrier for switching the magnetization is higher than the coupling strength and the thermal energy (Fig. S4b). In order to demonstrate that the nearest-neighbour coupling has switched from AP to P, the array was demagnetized again and an FM-like pattern was observed, confirming the change of coupling from AP to P (Fig. S4c). Therefore, experimentally, demagnetization of the array is essential to show the conversion of the voltage-controlled coupling.


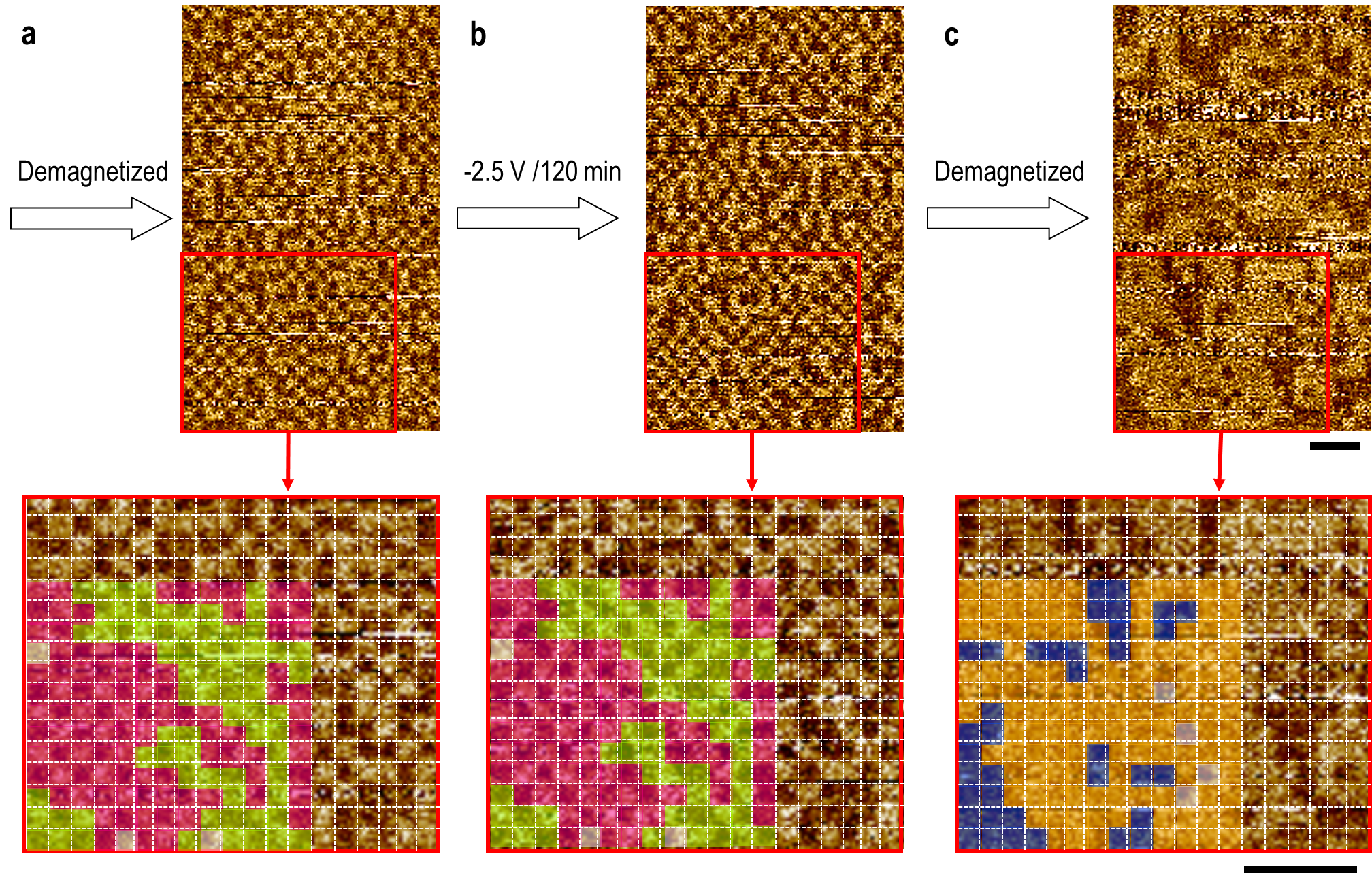


**Figure S4 | Demagnetized configurations following the electric gating. a**, MFM image of the magnetic configuration of the as-fabricated square-lattice array following demagnetization. **b**, MFM image of the magnetic configuration in the same area after applying a negative voltage of -2.5 V for 120 min. **c**, MFM image of the magnetic configuration in the same area following a second demagnetization. AFM-like domains are shaded in green and purple in the zoomed-in regions of **a** and **b**, and FM-like domains are shaded in yellow and blue in the zoomed in region of **c**. The bright and dark areas in the nanomagnet regions in the MFM images correspond to ↑ and ↓ magnetization, respectively. The scale bars are 1 µm.

**S3. Details of macrospin and semi-micromagnetic model**

In this section, we will first give a more detailed description of the macrospin model that was briefly introduced in the main text and shown in Fig. 3a. We then turn to a semi-micromagnetic model to quantitatively estimate the coupling strength.

In the macrospin model, due to the strong OOP magnetic anisotropy, **S**_1_ and **S**_2_ can only point either ↑ or ↓. The tilt angle *θ* of **S**_g_ is determined by minimizing the total energy. For AP alignment (**S_1_** = ↑ and **S_2_** = ↓), the energy can be written as:

$\begin{matrix} E_{\text{AP}}=-J_{\text{ex}}\left[\cos\theta+\cos(\pi-\theta)\right]-D_{\text{eff}}\left[\sin\theta+\sin(\pi-\theta)\right]-K_{\text{g}}V_{\text{g}}\cos^{2}\theta\\ \text{ }=-2D_{\text{eff}}\sin\theta-K_{\text{g}}V_{\text{g}}\cos^{2}\theta\end{matrix}$. (S6)

When *K*_g_*V*_g_ < -*D*_eff_, sin*θ*= -1 i.e., **S**_g_ = ← and *E*_AP_ = 2*D*_eff_. When *K*_g_*V*_g_ ≥ -*D*_eff_, sin*θ*= *D*_eff_/*K*_g_*V*_g_ and *E*_AP_ = - *D*_eff_^2^/*K*_g_*V*_g_ ‑ *K*_g_*V*_g_. For P alignment (**S**_1_ = ↑ and **S**_2_ = ↑), the energy can be written as:

$\begin{matrix} E_{\text{P}}=-J_{\text{ex}}\left[\cos\theta+\cos\theta\right]-D_{\text{eff}}\left[\sin\theta+\sin(-\theta)\right]-K_{\text{g}}V_{\text{g}}\cos^{2}\theta\\ \text{ }=-2J_{\text{ex}}\cos\theta-K_{\text{g}}V_{\text{g}}\cos^{2}\theta\end{matrix}$. (S7)

Similarly, when *K*_g_*V*_g_ < -*J*_ex_, cos*θ*= -*J*_ex_/*K*_g_*V*_g_ and *E*_P_ = *J*_ex_^2^/*K*_g_*V*_g_. When *K*_g_*V*_g_ ≥ -*J*_ex_, cos*θ*= 1 i.e., **S**_g_ = ↑ and *E*_P_ = -2*J*_ex_ -*K*_g_*V*_g_.


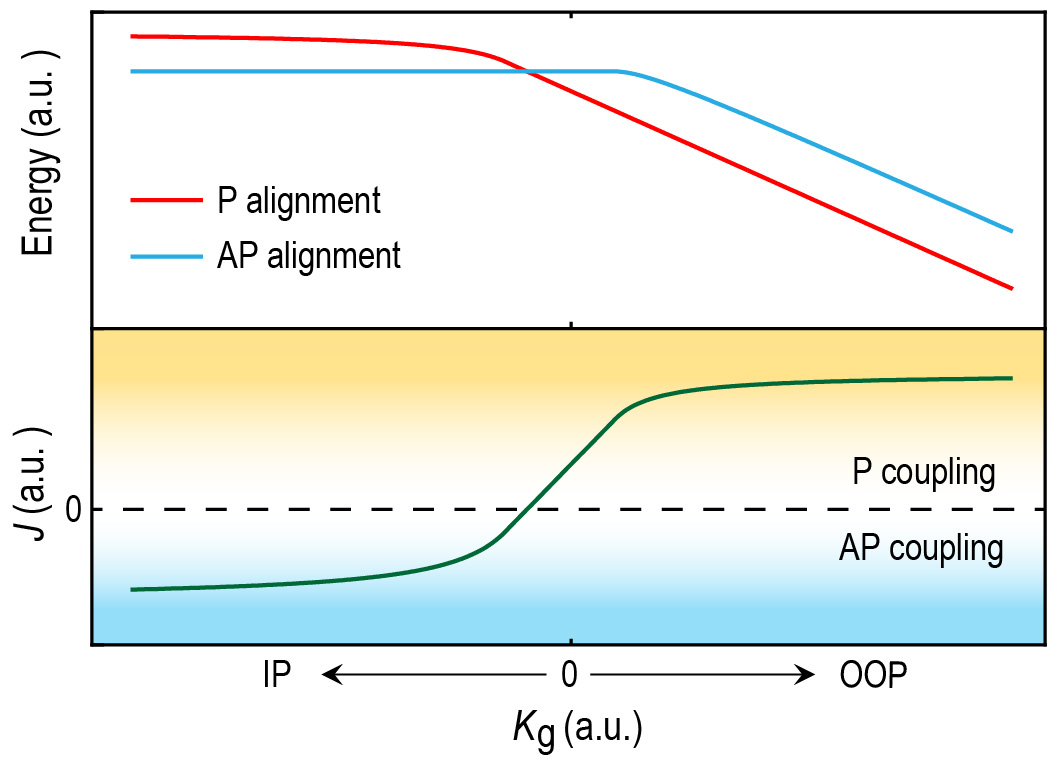


**Figure S5 | Energies for AP and P alignment, and coupling strength as a function of *K*_g_ obtained from the macrospin model with *J*_ex_ = 1.5 eV and *D*_eff_ = -1 eV.**

In Pt/Co, the antisymmetric exchange interaction is weaker than the symmetric exchange interaction, i.e. |*J*_ex_| > |*D*_eff_|. The energy curves for the AP and P configurations are shown in Fig. S5. By determining the difference in energy between AP and P alignment, we can obtain the strength of the coupling between **S**_1_ and **S**_2_. As discussed in the main text, if the gated region is strongly IP (*K*_g_ << 0), *J* ≈ *D*_eff_ < 0, whereas if the gated region is strongly OOP (*K*_g_ >> 0), *J* ≈ *J*_ex_ > 0, so providing an intuitive picture for the AP/P coupling conversion resulting from the *K*_g_-mediated competition between symmetric and antisymmetric exchange interaction.

Despite the fact that it is possible to explain the AP/P coupling conversion with the macrospin model, the “effective” interaction terms of *J*_ex_ and *D*_eff_ in Eq. 3 are not clearly related to the material parameters in real devices. To get closer to a real physical system, a semi-micromagnetic analytical model is developed on the basis of the macrospin model.


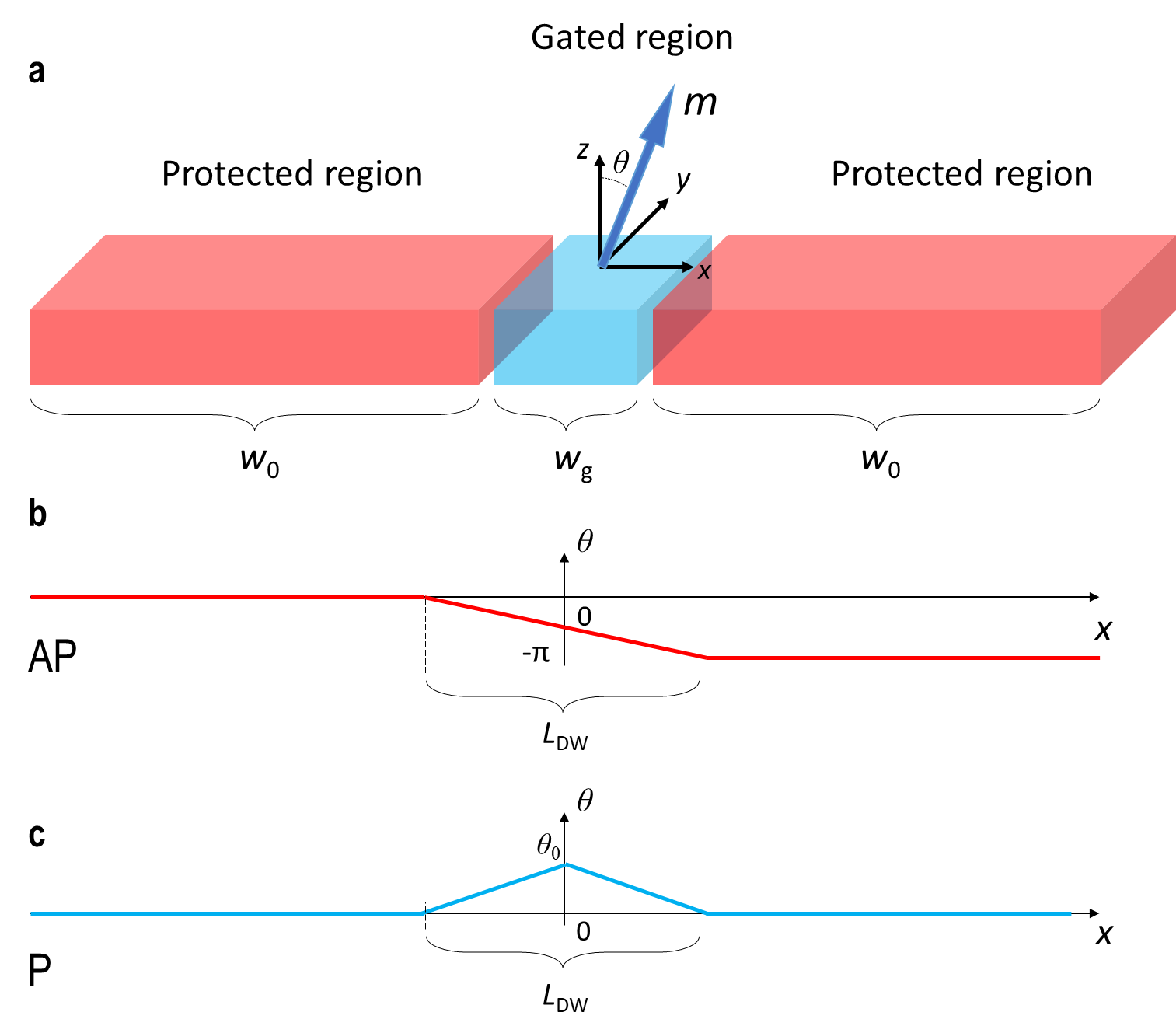


**Figure S6 | Semi-micromagnetic model. a,** Schematic of the basic element used for semi-micromagnetic model. **b** and **c**, Schematics of magnetization tilt angle *θ* for AP (**b**) and P (**c**) alignment of the magnetization in the neighbouring protected regions.

In the semi-micromagnetic model, the total energy including exchange energy, anisotropy energy and DMI energy, can be written as:

, (S8)

where *A*, *K* and *D* are the exchange energy constant, anisotropy constant and the DMI constant, respectively. *A* and *D* are constant throughout the magnetic regions, while *K* is different in the protected and gated regions. We denote the anisotropy constant within the gated region as *K*_g_ and as *K*_0_ for the protected region.

For simplicity, we assume the magnetization lies in *xz* plane and rotates linearly within domain walls. The structure of the basic element used for the model is shown schematically in Fig. S6a.

(i) For AP alignment (**S**_1_ = ↑ and **S**_2_ = ↓) (Fig. S6b), the boundary condition is $\left.\theta\right|_{x=-w_{0}-\frac{w_{\text{g}}}{2}}=0$ and $\left.\theta\right|_{x=w_{0}+\frac{w_{\text{g}}}{2}}=-\pi$. Considering the continuous magnetization rotation and the symmetry of the structure, the magnetization at the centre can be either ← or →. However, due to the left-handed chirality in Pt/Co, the magnetization at the centre prefers to be ← i.e., $\left.\theta\right|_{x=0}=-\frac{\pi}{2}$. Hence the magnetization can be written as:

$\hat{m}=[\sin\theta,0,\cos\theta]$, and

$\theta=\left\{\begin{matrix} 0;\text{ when }-\frac{w_{\text{g}}}{2}-w_{0}\leq x\leq-\frac{L_{\text{DW}}}{2} \\ -\frac{\pi}{L_{\text{DW}}}x-\frac{\pi}{2};\text{ when }-\frac{L_{\text{DW}}}{2}\leq x\leq\frac{L_{\text{DW}}}{2} \\ -\pi;\text{ when }\frac{L_{\text{DW}}}{2}\leq x\leq\frac{w_{\text{g}}}{2}+w_{0} \end{matrix}\right.$, (S9)

where *L*_DW_ represents the domain wall width.

Substituting Eq. S9 into Eq. S8, we obtain the following expression for the energy:

$\begin{matrix} E_{\text{AP}}=\int Adx[(\frac{\partial\hat{m}_{x}}{\partial x})^{2}+(\frac{\partial\hat{m}_{z}}{\partial x})^{2}]+\int-Kdx\hat{m}_{z}^{2}+\int-Ddx\left(\hat{m}_{z}\frac{\partial\hat{m}_{x}}{\partial x}-\hat{m}_{x}\frac{\partial\hat{m}_{z}}{\partial x}\right) \\ \text{ }=\int A\left(\frac{\partial\theta}{\partial x}\right)^{2}dx+\int-Kdx\cos^{2}\theta+\int-Ddx\left(\frac{\partial\theta}{\partial x}\right)=E_{\text{ex}}+E_{\text{an}}+E_{\text{DM}} \end{matrix}$ , (S10)

$E_{\text{ex}}=\frac{AS\pi^{2}}{L_{\text{DW}}}$,

$E_{\text{an}}=-2K_{0}w_{0}S-K_{\text{g}}w_{\text{g}}S+\frac{K_{0}L_{\text{DW}}S}{2}+\frac{L_{\text{DW}}S\sin\left(2\pi w_{\text{g}}/L_{\text{DW}}\right)}{2\pi}\left(K_{\text{g}}-K_{0}\right)$,

$E_{\text{DM}}=\pi DS$.

where *S* is the cross-sectional area.

When *K*_g_ < 0, and after the total energy minimization with respect to *L*_DW_, one finds: $L_{\text{DW}}=\pi\sqrt{\frac{2A}{K_{0}}}$. Since the gated region is narrow, we assume that *L*_DW_ > *w*_g_ and $\sin\left(2\pi w_{\text{g}}/L_{\text{DW}}\right)\approx2\pi w_{\text{g}}/L_{\text{DW}}$. Taking *L*_DW_ into account, the energy expression becomes

$E_{\text{AP}}=\pi S\sqrt{2AK_{0}}-2K_{0}w_{0}S-K_{\text{g}}w_{\text{g}}S+S\sqrt{\frac{A}{2K_{0}}}\sin\left(w_{\text{g}}\sqrt{\frac{2K_{0}}{A}}\right)\left(K_{\text{g}}-K_{0}\right)+\pi DS$. (S11)

When the OOP magnetic anisotropy in the gated region is relatively strong (*K*_g_ > 0), the domain wall will be fully located in the gated region i.e., *L*_DW_ < *w*_g_. The expression for the energy can then be written as:

$E_{\text{AP}}=\int AS\left(\frac{\partial\theta}{\partial x}\right)^{2}dx+\int-KSdx\cos^{2}\theta+\int DSdx\left(\frac{\partial\theta}{\partial x}\right)=E_{\text{ex}}+E_{\text{an}}+E_{\text{DM}}$, (S12)

$E_{\text{ex}}=\frac{AS\pi^{2}}{L_{\text{DW}}}$,

$E_{\text{an}}=-2K_{0}w_{0}S-K_{\text{g}}w_{\text{g}}S+\frac{K_{\text{g}}L_{\text{DW}}S}{2}$,

$E_{\text{DM}}=\pi DS$.

By minimizing the total energy with respect to *L*_DW_, we find: $L_{\text{DW}}=\pi\sqrt{\frac{2A}{K_{\text{g}}}}$. Then, introducing *L*_DW_ into the energy, we obtain:

$E_{\text{AP}}=\pi S\sqrt{2AK_{\text{g}}}-2K_{0}w_{0}S-K_{\text{g}}w_{\text{g}}S+\pi DS$. (S13)

(ii) For P alignment (**S**_1_ = ↑ and **S**_2_ = ↑) (Fig. S6c), the boundary condition is $\left.\theta\right|_{x=-w_{0}-\frac{w_{\text{g}}}{2}}=0$ and $\left.\theta\right|_{x=w_{0}+\frac{w_{\text{g}}}{2}}=0$. The magnetization can then be written as:

$\hat{m}=[\sin\theta,0,\cos\theta]$,

$\theta=\left\{\begin{matrix} 0;\text{ when }-\frac{w_{\text{g}}}{2}-w_{0}\leq x\leq-\frac{L_{\text{DW}}}{2} \\ \frac{2\theta_{0}}{L_{\text{DW}}}x+\theta_{0};\text{ when }-\frac{L_{\text{DW}}}{2}\leq x\leq0 \\ -\frac{2\theta_{0}}{L_{\text{DW}}}x+\theta_{0};\text{ when 0}\leq x\leq\frac{L_{\text{DW}}}{2} \\ 0;\text{ when }\frac{L_{\text{DW}}}{2}\leq x\leq\frac{w_{\text{g}}}{2}+w_{0} \end{matrix}\right.$. (S14)

According to the macrospin model, $\theta_{0}=\pm\frac{\pi}{2}$ when *K*_g_ << 0 and $\theta_{0}=0$ when *K*_g_ >> 0.

We first consider the case where *K*_g_ << 0 i.e., when the gated region is IP magnetized and$\theta_{0}=\frac{\pi}{2}$. Substituting Eq. S14 into Eq. S8, we obtain the following expression for the energy:

$E_{\text{P}}=\int AS\left(\frac{\partial\theta}{\partial x}\right)^{2}dx+\int-KSdx\cos^{2}\theta+\int DSdx\left(\frac{\partial\theta}{\partial x}\right)=E_{\text{ex}}+E_{\text{an}}+E_{\text{DM}}$, (S15)

$E_{\text{ex}}=\frac{AS\pi^{2}}{L_{\text{DW}}}$,

$E_{\text{an}}=-2K_{0}w_{0}S-K_{\text{g}}w_{\text{g}}S+\frac{K_{0}L_{\text{DW}}S}{2}+\frac{L_{\text{DW}}S\sin\left(2\pi w_{\text{g}}/L_{\text{DW}}\right)}{2\pi}\left(K_{\text{g}}-K_{0}\right)$,

$E_{\text{DM}}=0$.

After minimizing the total energy with respect to *L*_DW_, we find: $L_{\text{DW}}=\pi\sqrt{\frac{2A}{K_{0}}}$. Again, since the gated region is narrow, we assume *L*_DW_ > *w*_g_ and $\sin\left(2\pi w_{\text{g}}/L_{\text{DW}}\right)\approx2\pi w_{\text{g}}/L_{\text{DW}}$. Taking *L*_DW_ into the energy expression, we obtain:

$E_{\text{P}}=\pi S\sqrt{2AK_{0}}-2K_{0}w_{0}S-K_{\text{g}}w_{\text{g}}S+S\sqrt{\frac{A}{2K_{0}}}\sin\left(w_{\text{g}}\sqrt{\frac{2K_{0}}{A}}\right)\left(K_{\text{g}}-K_{0}\right)$. (S16)

We then consider the case of *K*_g_ >> 0 i.e., when the gated region is OOP magnetized and take $\theta_{0}=0$. Substituting Eq. S14 into Eq. S8, we obtain the following expression for the energy:

$E_{\text{P}}=\int AS\left(\frac{\partial\theta}{\partial x}\right)^{2}dx+\int-KSdx\cos^{2}\theta+\int DSdx\left(\frac{\partial\theta}{\partial x}\right)=E_{\text{ex}}+E_{\text{an}}+E_{\text{DM}}$, (S17)

$E_{\text{ex}}=0$,

$E_{\text{an}}=-2K_{0}w_{0}S-K_{\text{g}}w_{\text{g}}S$,

$E_{\text{DM}}=0$.

Hence,

$E_{\text{P}}=-2K_{0}w_{0}S-K_{\text{g}}w_{\text{g}}S$. (S18)

By determining the energy difference between the AP and P configurations, we obtain:

$E_{\text{AP}}-E_{\text{P}}=\pi DS<0$ when $K_{\text{g}}<<0$ (S19)

and

$E_{\text{AP}}-E_{\text{P}}=\pi S\sqrt{2AK_{\text{g}}}+\pi DS>0$ when $K_{\text{g}}>>0$. (S20)

Comparing this with the results obtained from the macrospin model, we obtain the relationship between the physical parameters *D* and *A*, and the “effective” interaction terms *D*_eff_ and *J*_ex_:

(S21)

. (S22)

The validity of these equations is confirmed by the good agreement of the coupling strength with that obtained from general micromagnetic simulations as described in main text. In particular, the magnitude of the AP coupling is given by: (*D* = ‑1.5 mJ/m^2^ and *S* = 150 nm × 1.5 nm), while the magnitude of P coupling is given by: , where *A* = 16 pJ m^−1^ and *K*_g_ = *M*_S_*H*_K_/2 = 0.9 MA m^−1^ × 608.4 mT/2). In addition, we find that the P coupling strength has a$\sqrt{K_{\text{g}}}$ dependence (Fig. 3b). Since the value of *K*_g_ tends to saturate at a certain value for *V*_G_ < 0, the coupling strength *J* for the P coupling should have an upper limit.

**S4. Micromagnetic simulations for different *K*_g_**

**
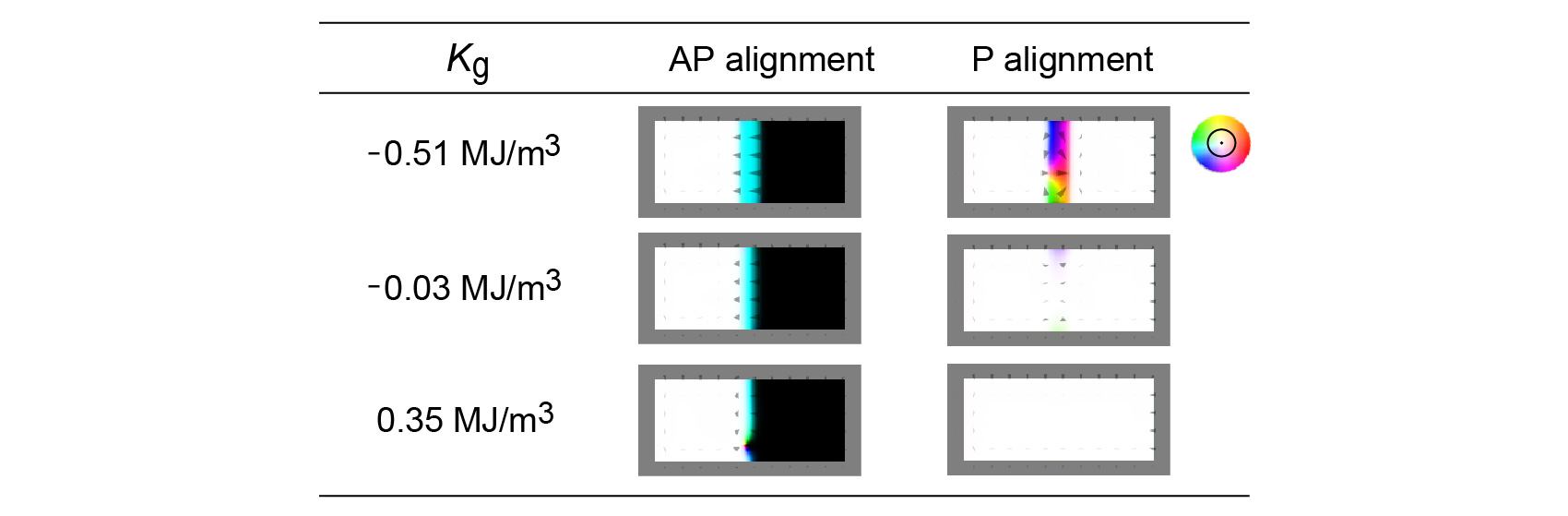
**

**Figure S7 | Snapshots of micromagnetic simulations for different *K*_g_.** The direction of the magnetization is indicated by the colour wheel, and white and black correspond to ↑ and ↓ magnetization, respectively.

**S5.** **Interplay between DMI and magnetic anisotropy**

In main text and Fig. 3, we present the relationship between coupling strength and magnetic anisotropy in the gated region determined from micromagnetic simulations. The effective OOP magnetic anisotropy *K*_eff_ used in the micromagnetic simulations is given by:

$K_{\text{eff}}=K_{\text{u}}-\frac{\mu_{0}M_{\text{S}}^{2}}{2}$, (S23)

where *K*_u_ and *μ*_0_ are the interfacial uniaxial magnetic anisotropy constant and the magnetic permeability of free space, respectively.

In Fig. S8, we present further results from the micromagnetic simulations, highlighting the interplay between DMI and magnetic anisotropy in the gated region. When *K*_g_ < 0, the energy of the system for AP and P alignment is almost the same in the absence of DMI, which supports the fact that DMI is responsible for AP coupling. With increasing DMI, the difference in energy for AP and P alignment increases when *K*_g_ < 0, indicating the enhancement of AP coupling. This leads to an increase in the critical *K*_g_ where the coupling is converted from AP to P. When *K*_g_ > 0, the energy of the system for AP alignment surpasses that for P alignment, resulting in a P coupling.

In the main text, we only consider the VCMA effect for the voltage control of the coupled nanomagnets. However, it has been reported that the DMI strength can be modified with electric fields and that the DMI decreases with decreasing OOP magnetic anisotropy since these two effects share the similar origin of spin-orbit coupling^4-7^. This may be partially the reason for the slight difference between experimental (-2.5 eV) and calculated values (-3.0 eV) of the AP coupling strength.


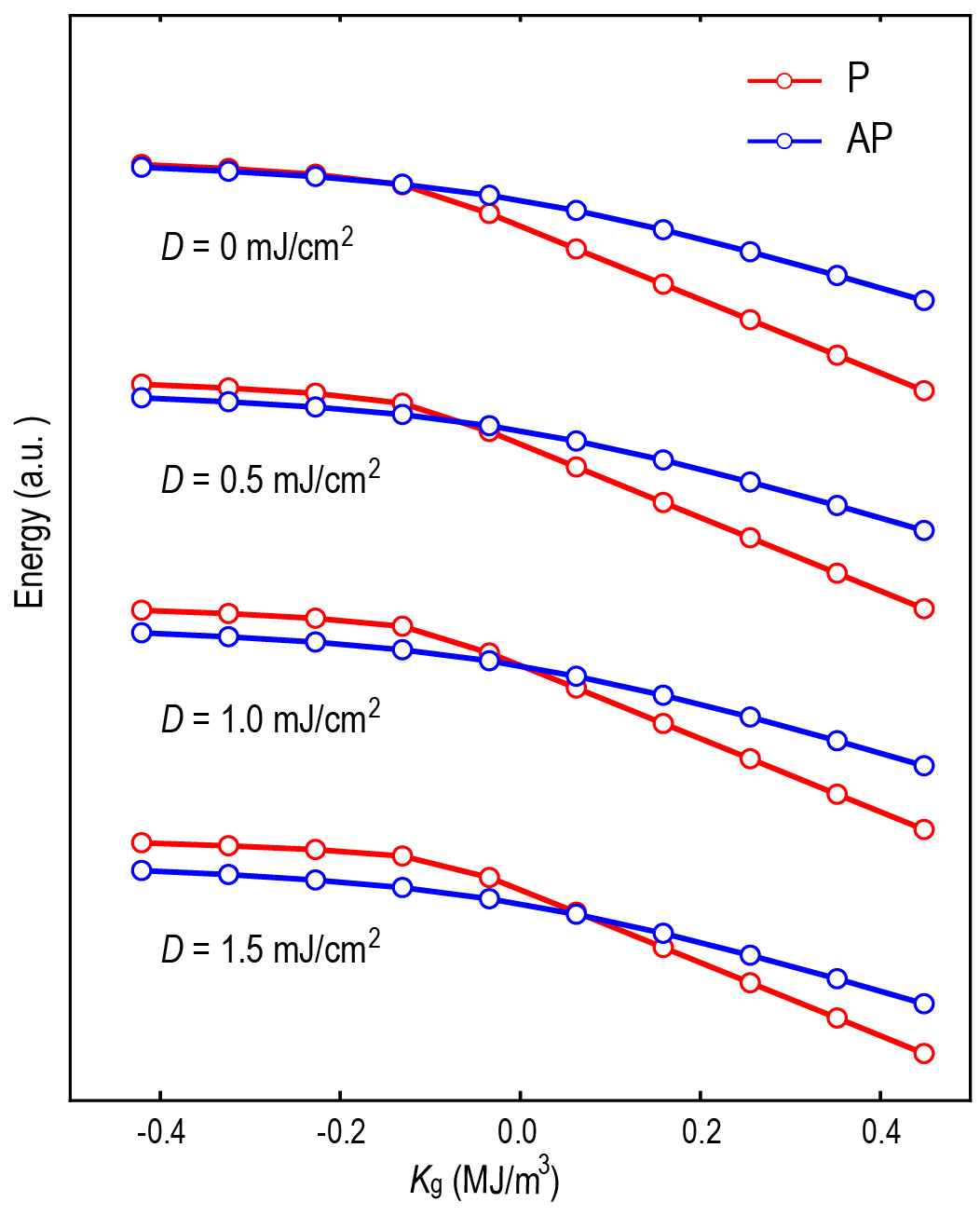


**Figure S8 | Energy of the system obtained from micromagnetic simulations for AP and P alignment as a function of *K*_g_ for different DMI values.**

**S6. Reliability of <*S_i_S_i_*_+1_> obtained from different chips and positions**

To illustrate the device-to-device reliability, the nearest-neighboring correlation function <*S_i_S_i_*_+1_> of the square lattice for 3 chips and 5 different devices per chip were measured as a function of the gate voltage (Fig. S9). The performance of the voltage-controlled magnetic coupling on changing the gate voltage is found to be robust with the trend in <*S_i_S_i_*_+1_> reproduced within <0.1 standard deviation on the same chip.


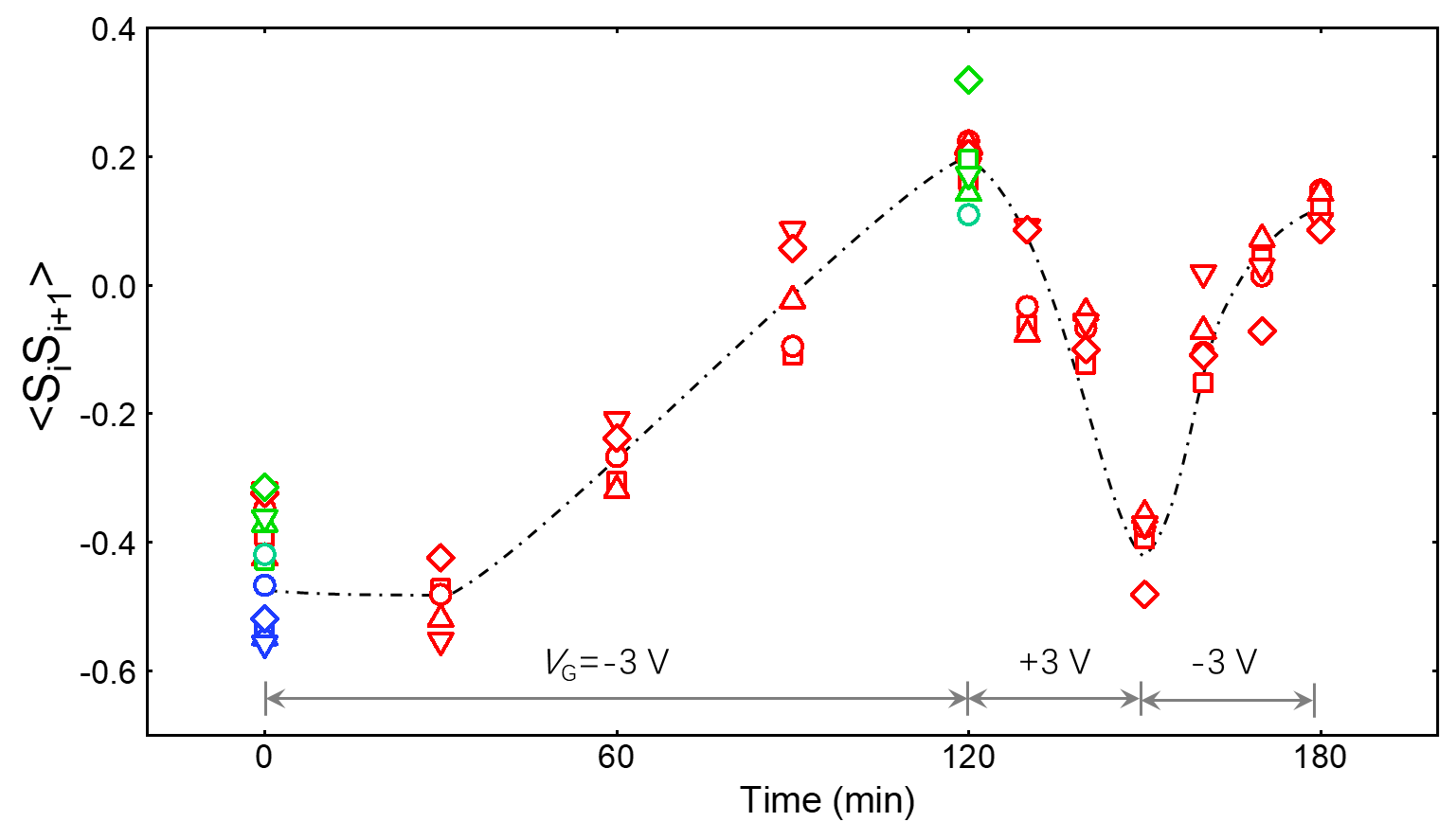


**Figure S9. <*S_i_S_i_*_+1_> as a function of gate voltage in square lattices with 15×15 nanomagnets obtained from 15 devices, with 5 devices fabricated on each of 3 different chips.** Red, green and blue colours indicate the results obtained from the 3 different chips, while the different symbols indicate the results obtained from the 5 different devices on the same chip.

**S7. Effect of the dipolar interaction**

Here, we determine the effect of the dipolar interaction in the Ising artificial spin ice. First, we estimate the contribution of the dipolar interaction for the basic element with two protected regions (Fig. 1a and 1b). A rough estimation of the energy of the dipolar coupling between the two protected regions can be obtained by considering two point-like dipoles placed at the centre of each element at a distance *r* from each other. In this case, the dipolar coupling *J*_dip_ is given by:

$J_{\text{dip}}=(E_{\uparrow\downarrow}-E_{\uparrow\uparrow})/2=-\frac{\mu_{0}m^{2}}{4\pi r^{3}}\approx-0.07\text{ eV}$ (S24)

with *m* = 3.0×10^-17^ A·m^2^ (for nanomagnet dimensions of 150 nm × 150 nm × 1.5 nm) and *r* = *w*_p_ + *w*_g_ = 200 nm, where *w*_p_ and *w*_g_ are the widths of the protected and gated region, respectively. This dipolar coupling is almost two orders of magnitude smaller than the measured coupling of 2.5 eV as well as the estimated exchange-induced coupling of 3.0 eV. Therefore, the effect of the dipolar interaction is negligible in the basic element.


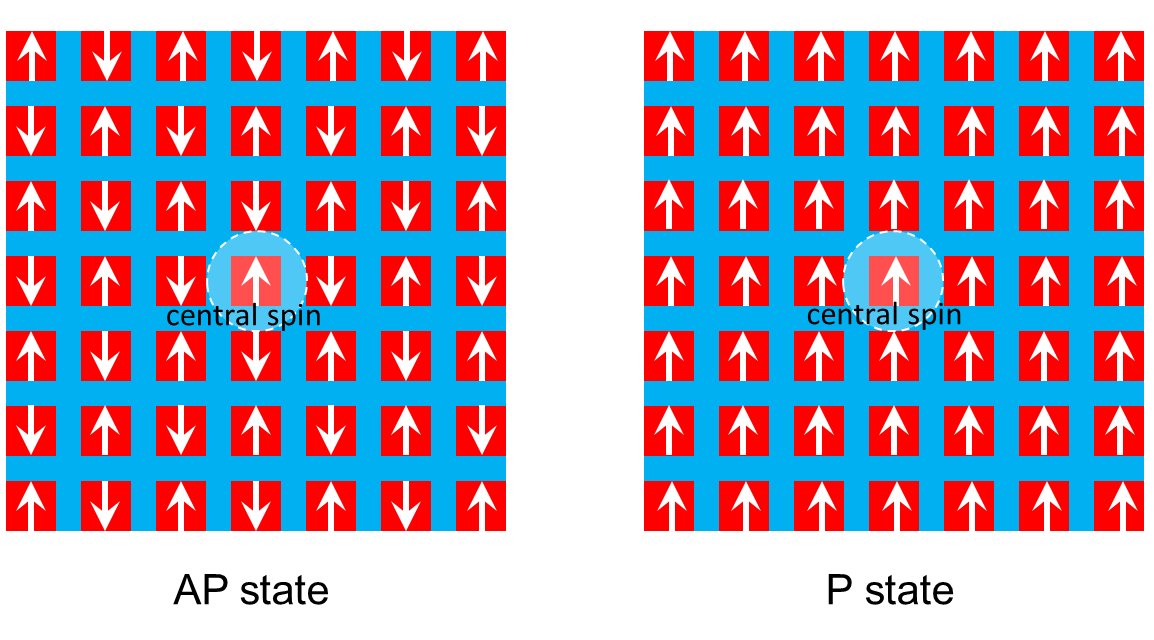


**Figure S10 | Schematics of magnetic configurations in the AP and P state used to estimate the effect of the dipolar coupling in an Ising artificial square ice.**

We now estimate the dipolar interaction in the extended Ising artificial square ices. For this, we consider the dipolar and exchange interactions in square lattice shown in Fig. S10. As the dipolar interaction is a long-range interaction, the energy associated with it needs to take into account the interactions from the surrounding macrospins. We evaluate the effect of the dipolar interactions by calculating the energy difference when flipping the central macrospin *S*_0_:

$\Delta E=E\left(S_{0}=\downarrow\right)-E\left(S_{0}=\uparrow\right)=\Delta E_{\text{dip}}+\Delta E_{\text{ex}}$. (S25)

where Δ*E*_dip_ and Δ*E*_ex_ are the change in the dipolar and exchange energies on flipping the central spin. For simplicity, we consider the case where the surrounding macrospins are in the ground state. For AP coupling, the ground state has AFM order, and the change in energy due to the dipolar interaction on flipping the central macrospin is:

$\Delta E^{\text{AP}}_{\text{dip}}=-\sum_{i} \frac{\mu_{0}m^{2}s_{i}}{2\pi r_{i}^{3}}\approx0.38\text{ eV}$ (S26)

where *s_i_* and *r_i_* represent the orientation of the *i*^th^ surrounding macrospins and the distance between the center and the *i*^th^ macrospin, respectively. The dipolar energy is summed over all surrounding macrospins in a square lattice with 100 × 100 nanomagnets similar to the experimental size. The dipolar interaction facilitates the formation of the AFM order. Due to the alternating up-down alignment of the magnetization for AP coupling, the energy change on flipping the central spin due to the dipolar interaction is small compared to Δ*E*_ex_ ≈ 8*J* ≈ 19.6 eV. For P coupling, the ground state has FM order, and the energy change due to the dipolar interaction on flipping the central macrospin is:

$\Delta E^{\text{P}}_{\text{dip}}=-\sum_{i} \frac{\mu_{0}m^{2}s_{i}}{2\pi r_{i}^{3}}\approx-1.29\text{ eV}$ (S27)

The dipolar interaction inhibits the formation of the FM order and, due to the uniform alignment of the macrospins in the P state, the energy difference induced by dipolar interaction becomes sizable.

In the experiment, we find that the strength of the AP and P coupling measured in the basic element is similar (shown by the similar exchange bias in Fig. 2d), while the correlation function <*S_i_S_i_*_+1_> for FM order is significantly smaller than that for AFM order in the extended structures (Fig. 4f). This could be due to the fact that the dipolar interaction becomes considerable in extended lattices and inhibits the formation of the FM order.

**S8. Programmable coupling configuration in a four-spin chain**

**
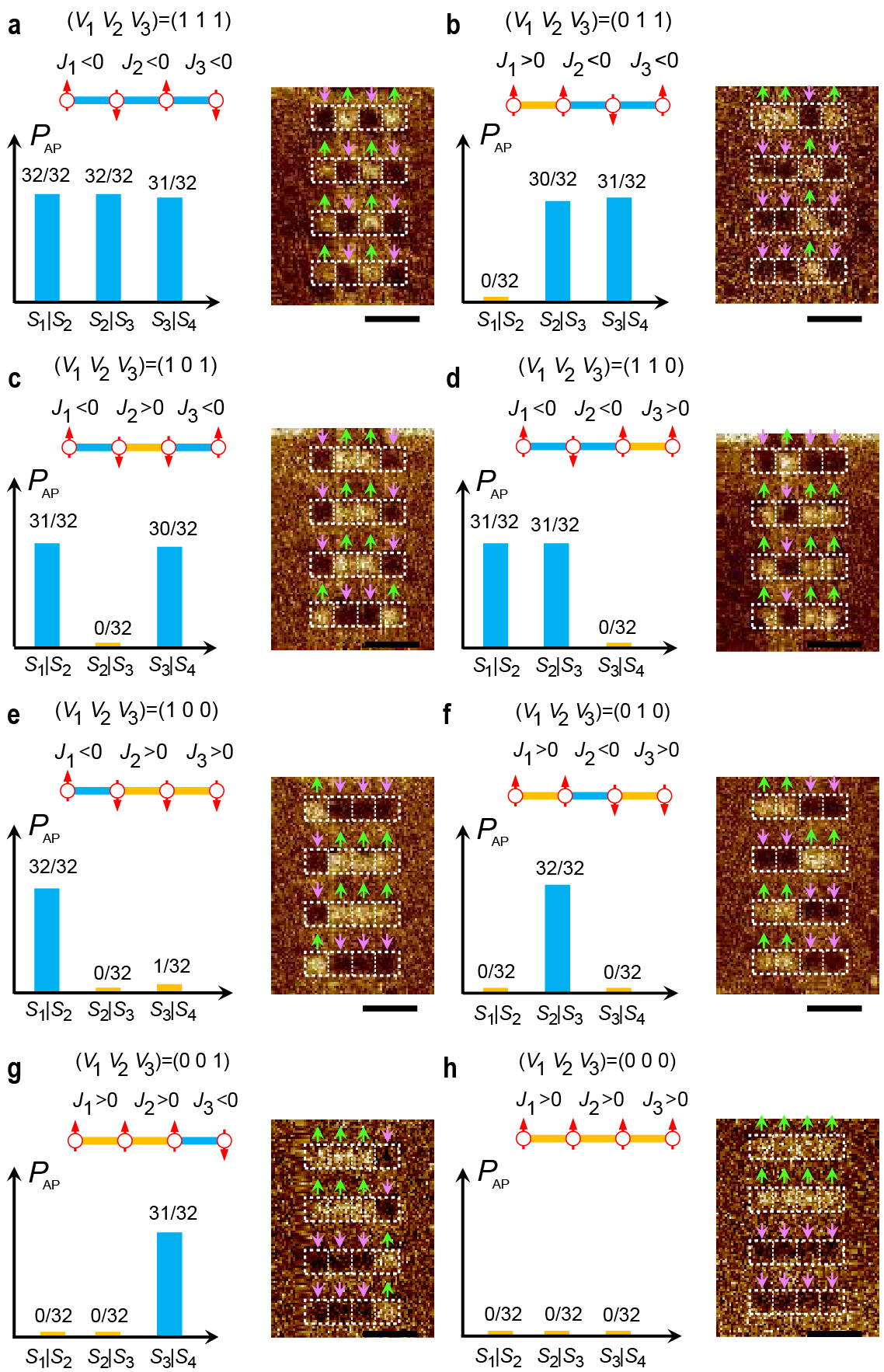
**

**Figure S11 |** **Programmable coupling configurations in a four-spin chain.** **a** to **h**, All 2^3^ = 8 coupling configurations that can be programmed using electric voltages. The applied voltages and corresponding coupling configurations, as well as one of the ground states, are shown. The blue and yellow connecting lines represent AP and P coupling, respectively. The percentages of AP alignment for the pairs of **S**_1_|**S**_2_, **S**_2_|**S**_3_ and **S**_3_|**S**_4_ after demagnetization are shown (left), illustrating the programmed coupling configuration. Each percentage is obtained from the measurement of 32 elements. The MFM images of four selected element structures are shown with green and purple arrows indicating the magnetization of ↑ and ↓ respectively (right). In the MFM images, the bright and dark areas in the nanomagnet regions correspond to ↑ and ↓ magnetization, respectively. In order to guarantee the AP/P conversion, the gate voltages are applied for 90 min. All scale bars are 500 nm.

**S9. Programmable Ising networks for the 8- and 10-vertex Max-Cut problems**


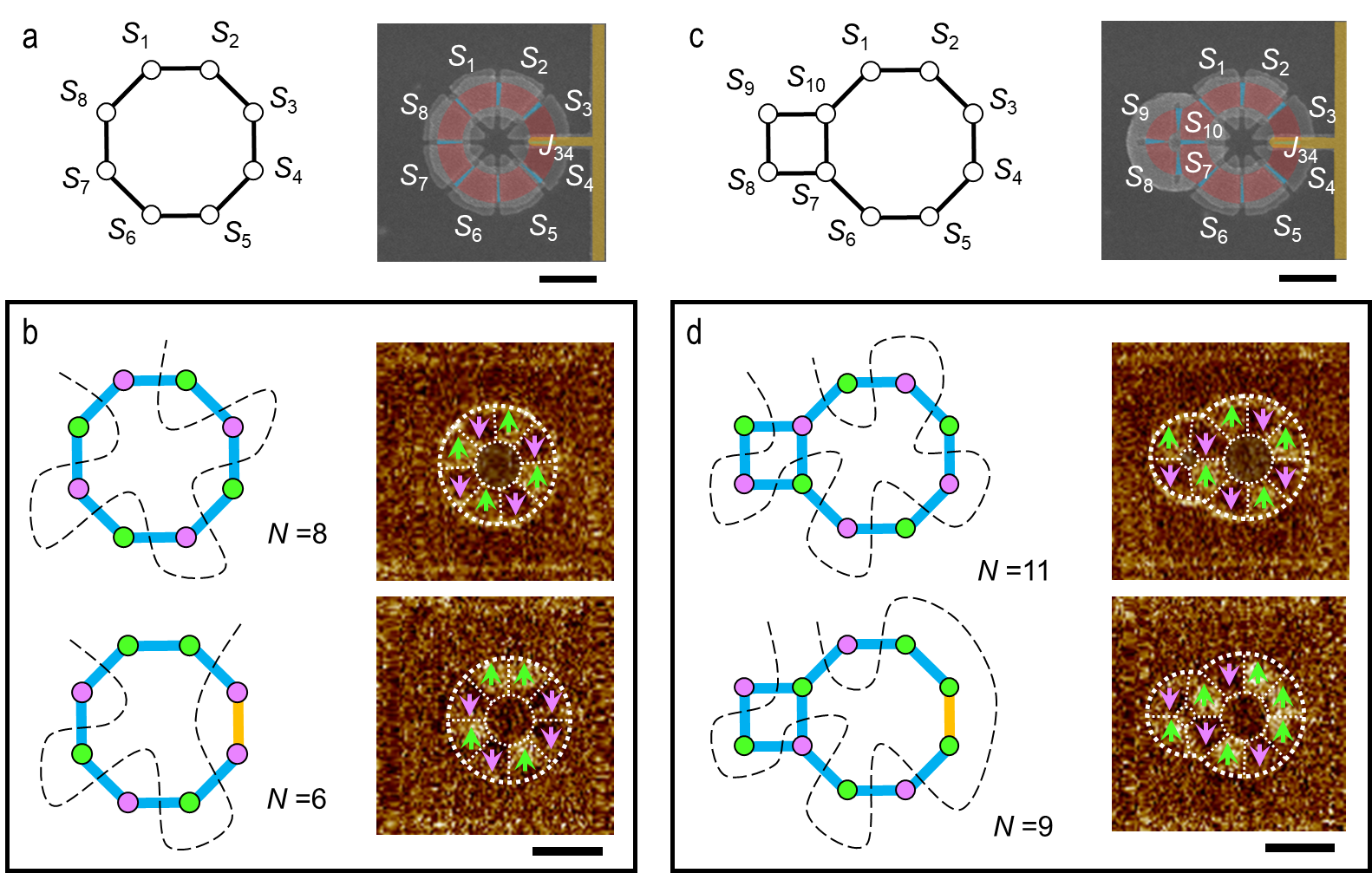


**Figure S12. Programmable Ising networks for the 8- and 10-vertex Max-Cut problems.** **a**, Schematic and coloured SEM image of a programmable 8-vertex Ising network. **b**, Solutions to Max-Cut problem obtained from MFM images of demagnetized devices for the cases when *J*_34_ is programmed to be AP (top) and P (bottom). **c**, Schematic and coloured SEM image of programmable 10-vertex Ising network. **d**, Solutions to Max-Cut problem obtained from MFM images of demagnetized devices for the cases when *J*_34_ is programmed to be AP (top) and P (bottom). The blue and yellow connecting lines in the schematics represent AP and P coupling. The black dashed line in each of the schematics indicates the cut lines separating vertices into two complementary sets (in green and purple), which is the solution to the Max-Cut problem with the corresponding weights. The bright and dark areas in the nanomagnet regions in the MFM images correspond to ↑ and ↓ magnetization, respectively, which is indicated with green and purple arrows. In the SEM images, red- and blue-shaded regions indicate the protected and gated regions, while the yellow-shaded region indicates the gate electrode. All the scale bars are 500 nm.

**S10. Hybrid MTJ/Ising network structure**

While we can exploit a nanomagnetic Ising network to map some combinatorial optimization problems to 2D Ising networks with only nearest-neighboring couplings, it is challenging to establish crossed connections beyond nearest neighbors due to the geometric limitation of the 2D physical structure. In order to realize a more general network, we propose a hybrid MTJ/Ising network in which the spin vertices can be electrically coupled via spin transfer torques in MTJs, so overcoming this geometric constraint.


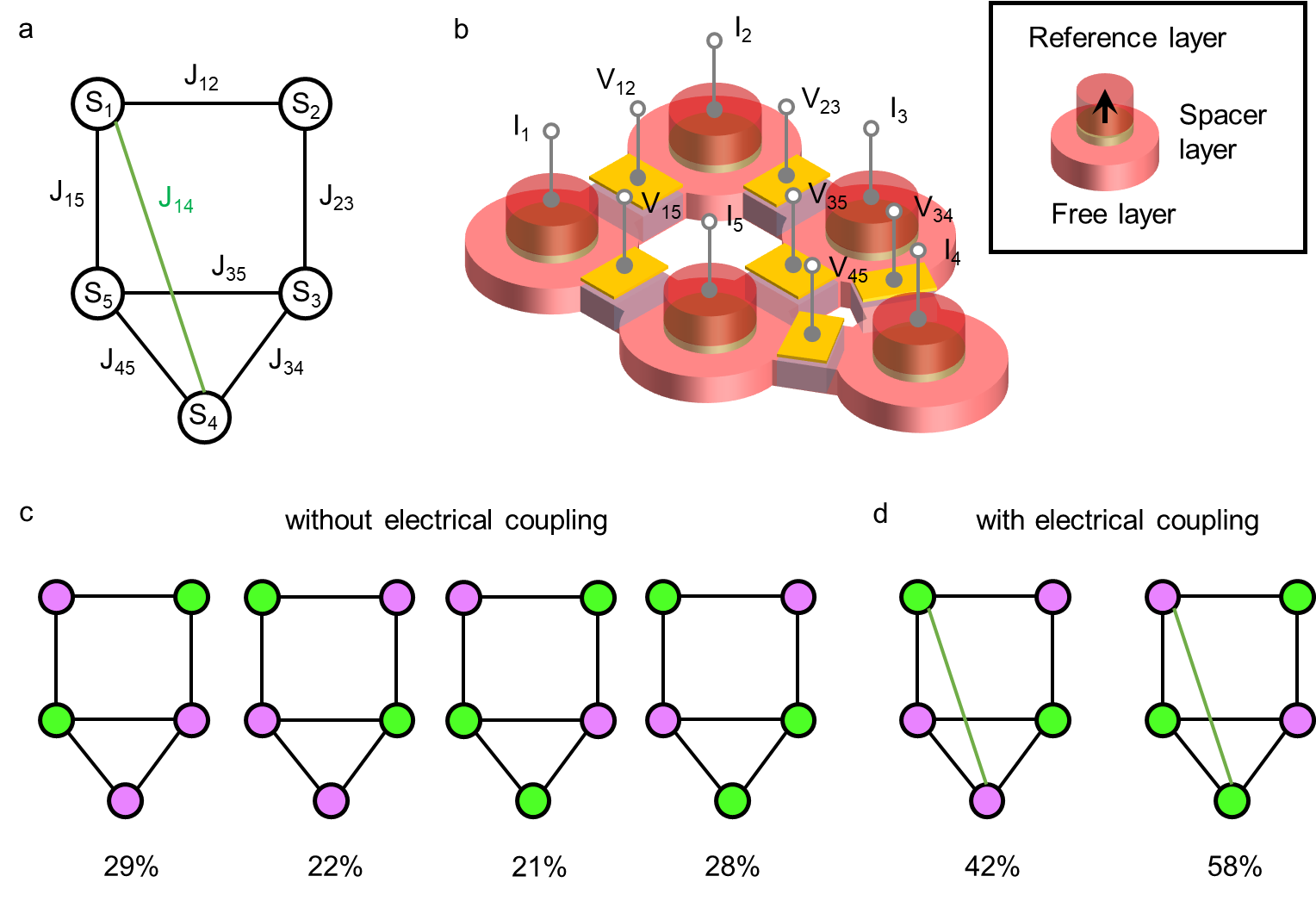


**Figure S13. Hybrid MTJ/Ising network structure for solving a complex Max-Cut problem. a,** Schematic of a 5-vertex Ising network. **b,** Schematic of the hybrid MTJ/Ising network structure. **c** and **d,** Simulation results of the ground state without (**c**) and with (**d**) electric coupling *J*_14_. The percentages of the ground spin state obtained from 100 simulation trials are indicated.

To demonstrate a complex network with crossed connections, we present in Fig. S13a and S13b, an Ising-like nanomagnetic network including 5 vertices (*S_i_*; *i*=1…5), 6 magnetic connections (*J*_12_, *J*_23_, *J*_34_, *J*_45_, *J*_35_, *J*_15_; *J_ij_* <0) and 1 electrical connection (*J*_14_ <0). The nanomagnetic structure is similar to that shown in Fig. 6, which also contains two rings with an even and odd number of vertices. MTJs are fabricated on each spin vertex, and can be used to read and write the magnetization of the underlying Ising element (free layer) via the tunnel magnetoresistance effect and the spin transfer torque (STT) effect, respectively (Fig. S13b). Each MTJ is addressed by a current *I_i_*. When *I_i_* is small, the STT effect is negligible and the magnetization of the Ising element (free layer) can be read via the tunnel magnetoresistance effect. When *I_i_* is large, the STT effect tends to switch the magnetization of the Ising layer parallel or antiparallel to the reference layer, depending on the polarity of *I_i_*. Assuming the magnetization in the reference layer to be↑, a positive (negative) *I_i_* gives an effective magnetic field pointing ↑ (↓) whose strength is determined by the magnitude of *I_i_*. In addition, the ionic gate structures are fabricated on each connection region (shown in yellow in Fig. S13b) and the gate voltage *V_ij_* is used to tune the magnetic coupling *J_ij_* between the vertices *S_i_* and *S_j_*.

Coupling *J*_14_ between vertices *S*_1_ and *S*_4_ is not possible in the 2D structure. Instead, we can couple vertex *S*_1_ and vertex *S*_4_ by applying electric currents through the MTJs on vertex *S*_1_ and vertex *S*_4_. The ground state of the Ising network is then obtained by applying the demagnetization protocol. The magnetization of *S*_1_ and *S*_4_ is read via the MTJ resistance with a small electric current. In addition, the electric currents

$I_{1}=I_{14}\text{sign}(S_{4})$ (S28)

and

$I_{4}=I_{14}\text{sign}(S_{1})$, (S29)

are injected to couple *S*_1_ and *S*_4_ via the STT effect. Here, *I*_14_ is the magnitude of the electric current corresponding to the coupling strength *J*_14_ and sign(*S_i_*) determines the polarity of the electric current. As *J*_14_ <0, *I*_14_ <0 and the electric currents *I*_1_ and *I*_4_ cause the vertices of *S*_1_ and *S*_4_ to be AP. For example, when *S*_1_ =↑, *I*_4_ =*I*_14_ <0 and *S*_4_ experiences an effective magnetic field pointing ↓, resulting in a current-induced AP coupling. This electrical procedure including magnetization reading and electrical coupling, is continuously repeated throughout the demagnetization process to accomplish the integration of magnetic and electrical couplings.

In order to verify the effectiveness of the hybrid MTJ/Ising network structure, we performed a simulation with the same macrospin model used for the square lattice. For the case of all magnetic couplings, the demagnetization process gives the four degenerate low energy spin states with approximately equal percentages as shown in Fig. S13c. In the presence of the electrical coupling *J*_14_, the electric currents *I*_1_ and *I*_4_ effectively couple the magnetization direction of *S*_1_ to that of *S*_4_. In the simulations, we set the time period of updating the electric currents to be 1 ms, which is faster than the response time of magnetic field in our experiment. The STT-induced effective magnetic field is set to match the coupling strength of the magnetic coupling. After the demagnetization process, the simulation yields the doubly-degenerate low energy spin state with approximately equal percentages (Fig. S13d).

Similarly, multiple electrical couplings can be incorporated to realize a more complex network, by adopting the methodology used for p-bit computation. For a more general and complex Ising network, the vertex *S_i_* has *N_i_* virtual couplings interacting with vertices *S^i^*_1_…*S^i^_Ni_*, and the electric current *I_i_* is given by:

$I_{i}=\sum_{j=1}^{N_{i}} I_{ij}\text{sign}(S_{j})$ (S30)

where *I_ij_* is the magnitude of the electric current corresponding to the coupling strength *J_ij_*. During the demagnetization protocol, the magnetization of two spin vertices that are electrically coupled is read via the MTJ resistance with a small electric current. Then the electric currents required to give “virtual coupling” are calculated and injected into the corresponding MTJ.

As shown above, in order to realize a complex Ising network with the functionality of programmability, the Ising network should contain (1) a gating structure for programmability and (2) an MTJ structure for the electric coupling. Both are compatible with state-of-art CMOS-back-end-of-line nanofabrication techniques. A similar hybrid structure of MTJs and coupled free layers has been demonstrated in previous experiments^8,9^. Furthermore, the minimum feature size in our nanomagnetic device, *i.e.*, the width of the gated region, is 50 nm, which can be produced with large-scale nanofabrication of magnetic devices with good device reliability^10^.

Despite the fact that the 11-node Max-Cut problem appears to be simple, the conventional CMOS-based approach to solve this problem is very complicated. In order to find the solution to the Max-Cut problem, one should calculate the weight values for all possible spin configurations and choose the configuration with the maximum weight value. For an 11-node Max-Cut problem, the number of possible spin configurations is 2^11^= 2048 and, in order to perform this procedure, the CMOS-based hardware should contain multiple electronic circuits to execute the required operations such as selection, addition, multiplication, comparison and memory.

Every functional circuit is composed of tens of transistors and can only perform sequential operations. Even for the CMOS-based approach that mimics the annealing process with virtual spin vertices, each virtual spin vertex consists of a spin memory circuit, an exclusive OR (EOR) circuit and a majority-vote circuit, which is constructed using hundreds of transistors^11^. Moreover, the virtual annealing process needs additional control circuits to synchronize all the spin vertices.

In comparison, the principle behind the layout in our nanomagnetic Ising network is to directly map the graphic network onto a physical structure, which is relatively straightforward. Here the relative position of the nanomagnets simply corresponds to the arrangement of the vertices in the network and the design of the nanomagnets (shape and placement) gives the coordination (or connection) number of the vertices. The advantages of our physically-coupled Ising network are the built-in reconfigurable magnetic coupling and the simplicity of the demagnetization protocol required to find the ground state. So, compared to CMOS-based hardware, our approach based on nanomagnetic Ising networks requires less electronic components and hence has a smaller size.

Moreover, our approach can be scaled up to have a large number of spin vertices. This is because the increase of vertex number in the Ising network only extends the nanomagnetic structure, while the device fabrication process and demagnetization procedure to obtain the ground state remain the same. In contrast, for the conventional approach constructed with CMOS-based hardware, the required time and energy consumption to find the solution increase exponentially with the increase of spin vertex number, as the Ising machine needs to run all the possible spin configurations to find the lowest energy state, with the total number of configurations given by 2*^N^* where *N* is the number of spin vertices. The total number of configurations becomes huge for a large number of spin vertices. For example, even for 15 spin vertices there are more than 3×10^4^ configurations.

**S11. Reconfigurable nanomagnet logic gates**

Taking advantage of the binary nature of the Ising elements, a logic operation can be regarded as the ground state of a 2D Ising network with specific boundary conditions. When adjusting the logic inputs that determine the spin orientation of some specific vertices, the ground state configuration of the logic output vertices gives the result of the logic operation encoded in the network. As the logic operation only requires nearest-neighbouring interactions, our nanomagnetic structure provides a general physical platform to construct arbitrary logic circuits. By incorporating the voltage-controlled lateral coupling, the tunable magnetic coupling in our nanomagnetic structure allows for run-time reconfigurable logic operations. To demonstrate this capability, we have created a controlled-NOT gate, which is a fundamental Boolean logic gate (Fig. S14a). The dual operation functions of NOT and COPY can be interchanged according to the polarity of the gate voltage (Fig. S14b and S14c). When applying an electric voltage “1” to the gate electrode, the coupling is set to be AP and the direction of output magnetization is opposite to that of the input magnetization, thus accomplishing the NOT operation. If an electric voltage “0” is applied, the coupling is set to be P and the direction of the output magnetization is the same as that of the input magnetization, accomplishing the COPY operation.


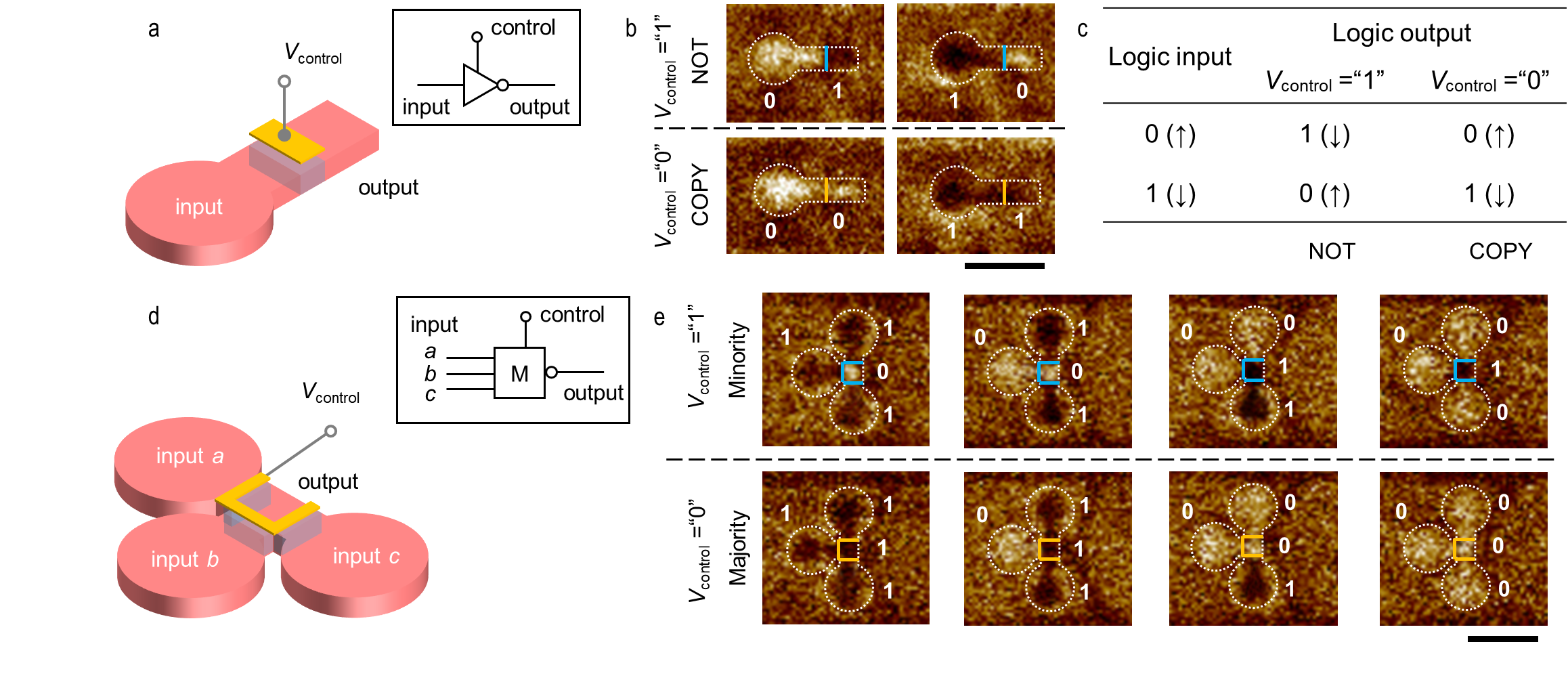


**Figure S14. Reconfigurable nanomagnetic logic gates.** **a**, Schematic of a controlled-NOT gate and corresponding logic circuit. **b**, MFM images of NOT operation for the AP coupling (top) and COPY operation for the P coupling (bottom). **c,** Truth table for the controlled-NOT gate. **d,** Schematic of a controlled-Majority gate and corresponding logic circuit. **e**, MFM images of Minority operation for the AP coupling (top) and Majority operation for the P coupling (bottom). In the schematics shown in **a** and **d**, red- and blue-shaded regions are the protected and gated regions, while yellow-shaded regions are the gate electrodes. The bright and dark areas in the nanomagnet regions in the MFM images correspond to ↑ and ↓ magnetization, respectively. The blue and yellow lines in the MFM images indicate the AP and P coupling. All scale bars are 500 nm.

Following the same principle, we can create a controlled-Majority gate (Fig. S14d), a functionally complete logic gate, and any Boolean function can be implemented using a combination of Minority gates. The output of the controlled-Majority gate depends on the relative alignments of the magnetization in the three inputs. As shown in Fig. S14e, if an electric voltage “1” (“0”) is applied to the gate electrode, the coupling is set to be AP (P) and the direction of the output magnetization is opposite (equal) to that of the majority of the three input magnetizations, accomplishing the Minority (Majority) operation. Therefore, our logic scheme has the capability of dynamic reconfigurability, which increases the logic functionality of a device without increasing the number of logic gates and leads to a more compact logic chip.

**References:**

1. Brown, W. F. et al. Thermal fluctuations of a single-domain particle. *Phys. Rev.* **130**, 1677 (1963).
2. Ke, X. et al. Energy minimization and ac demagnetization in a nanomagnet array. *Phys. Rev. Lett*. **101**, 037205 (2008).
3. Nisoli, C. et al. Effective temperature in an interacting vertex system: theory and experiment on artificial spin ice. *Phys. Rev. Lett*. **105**, 047205 (2010).
4. Schott, M. et al. The skyrmion switch: turning magnetic skyrmion bubbles on and off with an electric field. *Nano Lett*. **17**, 3006–3012 (2017).
5. Srivastava, T. et al. Large-voltage tuning of Dzyaloshinskii–Moriya interactions: a route toward dynamic control of skyrmion chirality. *Nano Lett*. **18**, 4871–4877 (2018).
6. Schott, M. et al. Electric field control of interfacial Dzyaloshinskii-Moriya interaction in Pt/Co/AlOx thin films. *J. Magn. Magn. Mater*. **520**, 167122 (2021).
7. Ba, Y. et al. Electric-field control of skyrmions in multiferroic heterostructure via magnetoelectric coupling. *Nature Commun*. **12**, 322 (2021).
8. Lyle, A. et al. Integration of spintronic interface for nanomagnetic arrays. *AIP Adv.* **1**, 042177 (2011).
9. Raymenants, E. et al. Nanoscale domain wall devices with magnetic tunnel junction read and write. *Nat. Electron.* **4**, 392–398 (2021).
10. Garello, K. et al. SOT-MRAM 300mm integration for low power and ultrafast embedded memories. In 2018 IEEE Symposium on VLSI Circuits 81-82 (IEEE 2018).
11. Takemoto, T. et al. A 2×30k-spin multi-chip scalable CMOS annealing processor based on a processing-in-memory approach for solving large-scale combinatorial optimization problems. *IEEE Journal of Solid-State Circuits* **55**, 145-156 (2019).
